# Engineering Approaches in Human Gamma Delta T Cells for Cancer Immunotherapy

**DOI:** 10.3389/fimmu.2018.01409

**Published:** 2018-06-26

**Authors:** Jonathan Fisher, John Anderson

**Affiliations:** University College London, London, United Kingdom

**Keywords:** gamma delta, chimeric antigen receptor, adoptive transfer, alpha beta T cells, cancer immunotherapy

## Abstract

Sharing both innate and adaptive immune properties, γδT cells are attractive candidates for cellular engineering. As the cancer immunotherapy field becomes increasingly busy, orthogonal approaches are required to drive advancement. Engineering of alternative effector cell types such as γδT cells represents one such approach. γδT cells can be modified using many of the techniques used in αβT cell engineering, with the added advantage of innate-like tumor recognition and killing. Progress has been made in T-cell receptor transfer to and from γδT cells as well as in a number of chimeric antigen receptor-based strategies. As the cancer immunotherapy field moves beyond repetitive iteration of established constructs to more creative solutions, γδT cells may offer an attractive chassis to drive anti-tumor responses that are not only broader, but also possess a more favorable safety profile.

## Introduction

Cellular engineering has offered many options for redirecting immune responses against cancer. In some cases, the clinical responses have been remarkable (1–5) but there are still many challenges to overcome. Broadly speaking, redirection of T-cell responses against specific tumor-associated antigens (TAAs) has been achieved in two ways: T-cell receptor (TCR) gene transfer or chimeric antigen receptor (CAR) expression. TCR gene transfer involves expression of a TCR derived from a tumor antigen-reactive T-cell (6–8). The TCR is typically derived from a tumor infiltrating lymphocyte or from *in vitro* antigen-stimulated blood. Chimeric antigen receptors (CARs) are more synthetic in nature and comprise an ectodomain that directly binds a cell surface molecule specific for the tumor and endodomains, which provide T cell signaling. The ectodomain is most commonly a single-chain variable fragment derived from a monoclonal antibody, and the endodomains usually include CD3ζ in combination with one or more costimulatory domains derived from molecules such as CD28 or 4-1BB (9, 10).

The majority of cellular engineering approaches have been applied to αβT cells, which are easy to expand and purify from peripheral blood. Notable attention has been given to αβT cells engineered to express second- and third-generation CARs against targets such as CD19 (2, 11–14) and CAR-T cells targeting CD19 recently received FDA approval for sale in the United States for the treatment of diffuse large B-cell lymphoma and acute lymphoblastic leukemia (ALL).

Engineering approaches that redirect immune cells to target single antigens *via* a CAR or MHC-presented TAA epitopes have limitations. TCR transfer depends on the ability to isolate a HLA-matched TCR against a processed antigen presented by tumor cells (10), and is susceptible to tumor immune-evasion strategies such as downregulation of MHC (15) or loss of redundant neo-antigens (16). Transferred TCRs against TAAs can also lead to unexpected side-effects due to cross-reactivity with unrelated peptides. One study targeting MAGE-3A with a HLA-A*01 restricted TCR led to fatal cardiotoxicity due to cross-reactivity with epitopes derived from the striated-muscle protein, titin (17), though a later study targeting the same molecule but using a different TCR construct did not generate this toxicity and led to objective partial responses in 9/17 patients (18). This difference may be explicable due to recognition of different epitopes, but highlights the potential for unexpected toxicity.

Chimeric antigen receptors remove the need for HLA-matching and antigen presentation on tumor MHC by bypassing the αβTCR entirely, but antigen selection presents a challenge. CAR-T cells target both healthy and tumor cells expressing their cognate antigen (10); for example, anti-CD19 CARs kill CD19^+^ ALL as well as healthy CD19^+^ B-cells (19). In the context of CD19, B-cell aplasia is considered an acceptable cost, but targeting of other antigens such as carbonic anhydrase IX or ErbB2 has led to unexpected and sometimes fatal toxicity (albeit only at very high T cell dose in the case of ErbB2) (20, 21). Furthermore, the specificity of CAR-targeting provides a prime opportunity for immune-evasion through antigen loss, which has proven to be a particular issue in anti-CD19 CAR-T therapy (22).

Use of alternative cell types in cancer immunotherapy is not a novel concept. Adoptively transferred allogeneic NK cells or cytokine-induced killer cells have shown clinical efficacy against metastatic melanoma (23), renal cell carcinoma, acute myeloid leukemia, and Hodgkins lymphoma (24). While engineering of these cell types has lagged behind that of conventional αβT cells, CAR transduced NK cell lines have been successfully directed against CD19 (25), CD20 (26), the disialoganglioside GD2 (27), ErbB2 (28), and other TAAs (29). NK cell specificity to tumors has been enhanced using exogenous constructs such as bispecific antibodies that enhance or manipulate the synapse between NK cell and target (30). NKT cells expressing CARs have also been developed (31). Such modified NKT cells targeting the ganglioside GD2 are about to enter phase I trials in patients with neuroblastoma (clinical trial ID NCT03294954). This range of approaches demonstrates the feasibility of using effector cells with an innate immune phenotype, possessing broader tumor recognition potential.

## Properties of γδT Cells

*In vitro* and *in vivo*, γδT cells exhibit potent anti-tumor responses suggesting natural roles in tumor control and potential for therapeutic exploitation (32–35). Of particular interest, a recent correlation between the molecular profile of the tumor immune microenvironment and prognosis in over 5,000 tumor samples indicated that the presence of infiltrating γδT cells was the strongest predictor of positive outcome (36).

γδT cells comprise only 1–10% of circulating T-cells (37), diverging from αβT cells in the thymus, with lineage commitment completed by the DN3 stage of thymic development (38). The dynamics of the γδT cell repertoire during fetal development and later adult life are complex, and while initial evidence suggested that the Vγ9Vδ2 subset, being small at birth (39), expanded purely in response to environmental pathogens, Dimova et al. showed that effector Vγ9Vδ2^+^ cells make up the bulk of the γδT cell repertoire in second trimester fetuses. This population contracts and loses its dominance toward full gestation, when Vγ9^−^Vδ1^+^ subsets predominate (40). These results were corroborated by Ravens et al. who used next-generation sequencing of the γδTCR repertoire in cord blood to reveal higher proportions of Vγ2–5 and Vδ1, 3 and 5 TCR chains compared to healthy adult circulation (41). Later in life, while adult human peripheral γδT cells expressing Vγ2–5, 8–9, and Vδ1–8 chains (42) can all be detected in peripheral blood of healthy donors and cancer patients (37), Vγ9Vδ2^+^ cells predominate in the circulation, and the age-related extrathymic increase in circulating Vγ9Vδ2^+^ proportions (39, 43) is well documented. Interestingly, this trend shows geographical variation; γδTCR repertoires of individuals from sub-Saharan Africa show greater enrichment of Vδ1^+^ cells compared to that of Caucasians living in Europe or America. This difference is not linked to malaria exposure and raises the possibility that the circulating γδTCR repertoire is shaped by environmental factors such as the endemic microbiome (43, 44).

Human Vγ9Vδ2^+^ T cells have been subjected to closer analysis than other subsets. They respond to targets with a high phosphoantigen burden, associated with malignant transformation and disordered EGFR signaling (45, 46). Importantly, this recognition is not dependent on peptide epitopes bound to MHC, distinguishing Vγ9Vδ2 T cells from αβT cells (47). Isopentenyl-5-pyrophosphate (IPP), a phosphoantigen by-product of the mevalonate pathway of cholesterol biosynthesis is the prototypic phosphoantigen in the context of human Vγ9Vδ2 T cell–tumor interactions (48, 49), though other phosphoantigens such as bromohydrin pyrophosphate and the microbially derived (*E*)-4-Hydroxy-3-methyl-but-2-enyl pyrophosphate have much lower EC_50_ values for Vγ9Vδ2 T cell activation (50). Because IPP production can be enhanced using aminobisphosphonates, Vγ9Vδ2^+^ T cells can be easily expanded from the blood of healthy donors and cancer patients using inexpensive and well-validated compounds in combination with low-dose IL-2 (50). Aminobisphosphonates inhibit the mevalonate pathway enzyme farnesyl pyrophosphate synthase, which is downstream of IPP and leads to its accumulation (51, 52). The approach allows production of large numbers of highly purified Vγ9Vδ2^+^ T cells using a relatively simple protocol (37, 53).

The precise mechanism of Vγ9Vδ2 TCR stimulus is still being clarified. There is a high degree of CDR3 sequence homology between TCR chains from fresh Vγ9Vδ2^+^ γδT cells, those expanded using aminobisphosphonates and those which expand in response to co-culture with microbes such as *Escherichia coli* (54). There is also homology in Vδ chain CDR3 regions between cells from unrelated individuals following phosphoantigen exposure (37). These factors reinforce the evidence that the Vγ9Vδ2 TCR responds to a ligand held in-common across donors. While previous reports have implicated F1-ATPase as the ligand (55, 56), strong recent evidence points to butyrophilin 3A1 (BTN3A1) (57, 58), which is stabilized in the membrane and undergoes a conformational change when its intracellular 30.2 domain is bound by IPP.

γδT cells also receive inputs from multiple co-stimulatory receptors and receptors usually associated with NK cells (59, 60), such as NKG2D (61), DNAM-1 (62), and Fcγ receptors, such as FcγRIII (CD16) (34, 63). Consequently, Vγ9Vδ2^+^ T cells exhibit NK-cell like properties of potent antibody-dependent and independent cytotoxicity (34, 53, 63). Less is known about the ligands of non-Vγ9Vδ2 γδTCRs, perhaps due to the diversity of targets and their MHC-independent activity. Numerous ligands have been identified but a clear pattern has not yet emerged, for example, Vδ1^+^ γδT cells have been shown to have against cells expressing lipids, such as CD1c (64), CD1d-sulphatide (65), CD1d-α-GalCer (66), but also against the MHC-associated molecules MICA (67) and MICB (68).

## TCR Gene Transfer in the Context of γδTCR^+^ Cells

Transfer of specificity through the transfer of murine α and β TCR genes was first used to target the hapten molecule, fluorescein (69), an approach which has subsequently been used to redirect αβT cell immunity against antigens from viral (70) and tumor (71) targets, notably in highly immunogenic tumors, such as melanoma.

**Figure 1:**
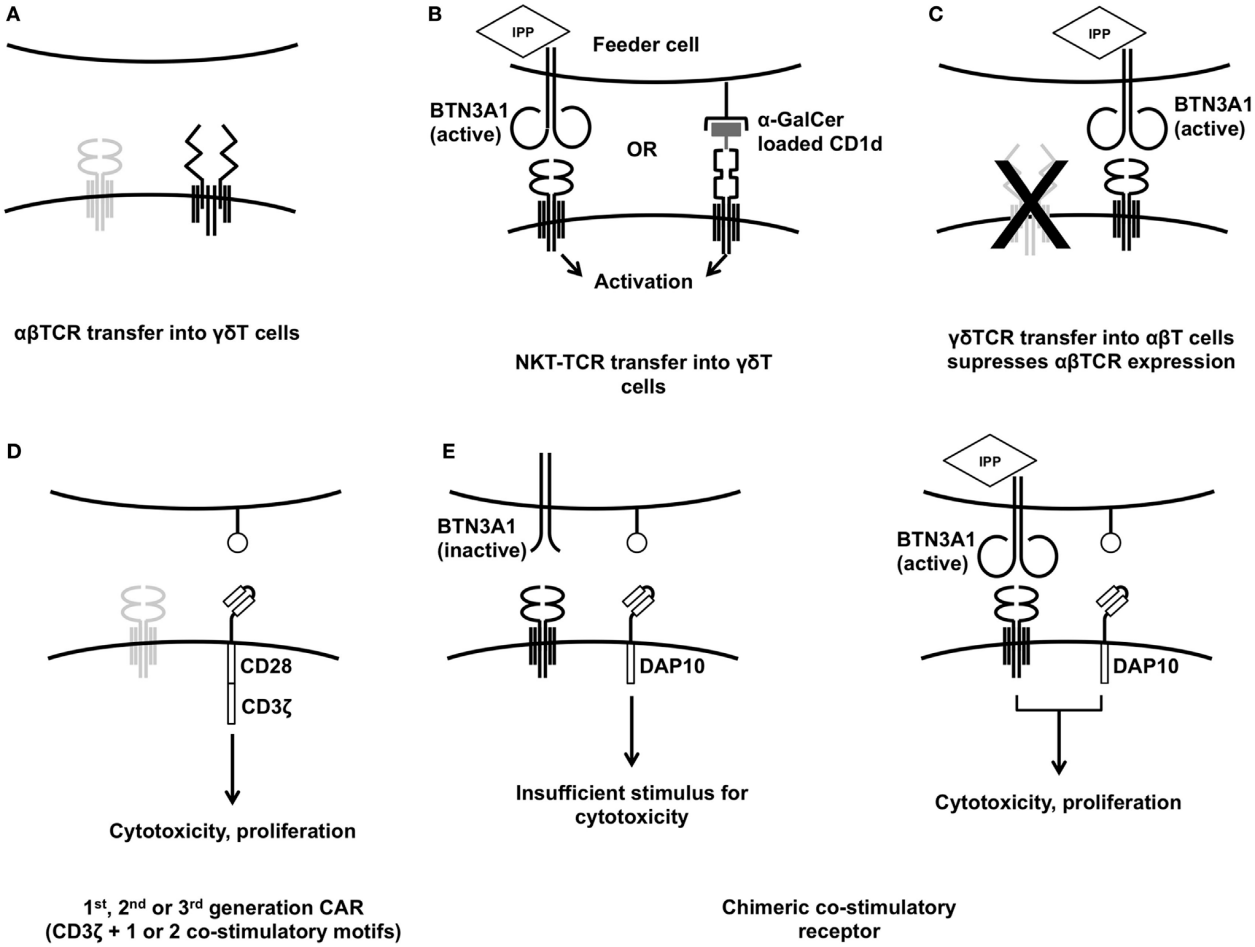
Established strategies for engineering γδT cells. Transferring specific αβT-cell receptor (TCR) into γδT cells **(A)** gives greater control of the final cell product and is one strategy to avoid TCR mis-pairing. Transferring NKT-TCRs into γδT cells **(B)** allows them to be activated for cytotoxicity using feeder cells treated with αGalCer or zoledronic acid. Transfer of γδTCRs into αβT cells has been used to impart broader anti-tumor reactivity **(C)**. Anti-tumor cytotoxicity can be enhanced using conventional first-, second-, or third-generation chimeric antigen receptors **(D)** but the innate tumor recognition provided by the γδTCR can also be harnessed to deliver a more tumor-specific response **(E)**.

Transferring a new αβTCR gene construct into an αβT cells runs the risk of TCR chain mis-pairing unless the endogenous α and β chains are suppressed (72). Mis-pairing can lead to inefficient expression of the novel construct and may lead to the generation of self-reactive TCR clones leading to off-target toxicity (73). Using murine constant regions or altering arrangement of cysteines in the transferred TCRs prevents this (74). While there is a risk of the host mounting an anti-murine immune response with subsequent reduction in immunotherapeutic efficacy, this has not been seen in practice, and many leading groups favor murinized TCRs. One study in which 23% of patients developed anti-murine-TCR antibodies showed that these anti-murine responses had no effect on clinical outcome (75). Were an anti-murine response to be of concern, however, an alternative allowing use of entirely human TCRs is to use γδT cells as the substrate for gene transfer, as the γ and δ TCR chains do not mis-pair with transferred α or β chains (Figure 1A). Dorrie et al. (76) demonstrated that γδT cells could be induced to express a HLA-A*0101 restricted αβTCR targeting a peptide derived from an adenovirus hexon protein. Engineered γδT cells produced more IFNγ and TNFα than CD8^+^ αβT cells expressing the same TCR and had equivalent cytotoxicity against autologous adenovirus-infected dendritic cells. Similar antigen-specific cytokine release was demonstrated by Harrer et al. when γδT cells expressing a gp100/HLA-A2 restricted αβTCR were exposed to gp100^+^ melanoma cells (77).

Some researchers have highlighted the restrictions inherent in αβTCR gene transfer, in particular the restriction to particular HLA types and the possibility of antigen-negative escape variants (16). Transfer of TCRs derived from invariant natural killer T (iNKT) cells into γδT cells and transfer of γδTCRs into αβT cells (78) have both been used to overcome this. In humans, iNKT cells express the nearly invariant TCR encoded by Vα24Jα18 which responds to glycolipids presented on the HLA-class-I-like molecule CD1d. Like Vγ9Vδ2 TCR activation, this response is not MHC restricted (79). γδT cells expressing TCRs derived from iNKT cells can be stimulated by co-culture with either zoledronic acid treated HEK293T cells or HEK293T cells pulsed with the exogenous glycolipid α-galactosylceramide (α-GalCer, Figure 1B). Both stimulations led to enhanced cytotoxicity against the CD1d^−^ leukemia cell line K562 (79). Continuing on the theme of using MHC-unrestricted TCRs, γδTCR gene transfer into αβT cells (Figure 1C) has yielded exciting results. The Vγ9Vδ2 TCR clone G115 (80) was expressed in αβT cells by Marcu-Malina et al. (78). They demonstrated that both the were required for either to be detected, indicating that mis-pairing with endogenous α or β chains was not occurring. The γδTCR-expressing αβT cells showed similar functional properties to “native” Vγ9Vδ2 cells including cytotoxicity against the Daudi cell line, release of TNFα and IFNγ, enhancement of cytotoxicity following target pre-treatment with aminobisphosphonates, and the ability to induce dendritic cell maturation. Vγ9Vδ2 transduced αβT cells showed a surprising lack of alloreactivity, linked to a downregulation of their endogenous αβTCRs (78), and were able to mount responses against a broad panel of tumor cell lines. This lack of γδT cell alloreactivity against non-transformed cells is corroborated by other *in vitro* data on both Vδ1^+^ and Vγ9Vδ2^+^ γδT cells (37).

Though the Vγ9Vδ2 TCRs derived from different T cell clones show varying anti-tumor responses, linked to small differences in the γ9 and δ2 CDR3 regions; no correlation was found between the expression of NKG2D, CD158a, NKAT-2, or NKB-1 and anti-tumor reactivity (81). This suggests that altering the functional avidity of interaction between BTN3A1 and the Vγ9Vδ2 TCR is a rich area for optimization. Using CD4^+^ αβT cells as the recipient cells, Gründer et al. performed alanine scanning between positions δ2-G115_L109_ and δ2-G115_T113_ and between γ9-G115_E108_ and γ9-G115_E111_, to demonstrate that the length and sequences in these CDR3 regions were critical for ligand interaction, with particular importance being placed on γ9-G115_A109_, in addition to the J-region residues δ_109_ and δ_117_ (81).

Such detailed knowledge of Vγ9Vδ2 avidity means that highly optimized γδTCRs can be expressed in more readily available αβT cells. γδTCR-engineered αβT cells prevented tumor growth in an immunodeficient (irradiated Rag^−/−^γc^−/−^) murine model of Burkitt lymphoma (Daudi) and multiple myeloma (OPM2) and also protected mice who had responded to initial treatment from re-challenge with OPM2 performed 120 days after the first tumor and T-cell injection (82). The downregulation of the αβTCR in the transduced cell population allows for facile selection of cells by αβTCR depletion, rather than positive selection of the transduced cells. This “untouched” cell product does not require co-expression of a marker gene and can be processed using pre-existing αβT cell depletion techniques currently used before some bone-marrow transplants, making it highly amenable to GMP-compliant manufacture.

## γδT Cells Expressing CARs

While harnessing the innate potential of the γδTCR is a highly attractive option, manipulating cellular behavior in an antigen-specific manner using CARs (Figure 1D) remains one of the mainstays of modern immunotherapeutics. Compared to the substantial body of literature on αβT cells expressing CARs, there are relatively few reports of CAR-γδT cells. First described in 2004 (83), γδT cells expressing a first-generation CAR-targeting GD2 (14.G2aζ) which is expressed on the surface of neuroblastoma and Ewing sarcoma cells (84, 85) showed enhanced antigen-specific tumor reactivity. Following co-culture with the GD2^+^ neuroblastoma cell line LAN-1, 14.G2aζ^+^Vγ9^+^ cells showed greater production of the Th1 cytokine IFNγ compared to non-transduced zoledronate expanded 14.G2aζ^−^Vγ9^+^ γδT cells. This effect was mirrored in the expression of the T-cell activation marker CD69, which also upregulated the presence of the tumor cells. In the absence of GD2^+^ cells, 14.G2aζ^+^Vγ9^+^ γδT cells showed only 1.5 ± 0.5% IFNγ^+^CD69^+^ but following co-culture with GD2^+^ LAN-1 targets this rose to 33 ± 3%. Background production of IFNγ by non-transduced effectors exposed to LAN-1 was low (5.7 ± 1.2%). Similar results were seen when γδT cells expressing the CD19ζ CAR were co-cultured with CD19^+^ cell lines Daudi, Raji, and Reh (83), with substantial increases in target-dependent IFNγ production by mixed populations of CD19ζ^+/−^ γδT cells. While Daudi is known to engage the γδTCR and is highly susceptible to γδT cell-mediated killing in its own right, Raji is usually considered to be a γδT cell resistant cell line (86), and it was in this model that the highest IFNγ production was seen, suggesting that CAR expression could overcome some of the immune-escape mechanisms shown by the target cells.

Since the publication of the work of Rischer et al. (83), progress in immunotherapy using adoptively transferred γδT cells has focused on the expansion of un-engineered γδT cells (32, 35). Whereas earlier studies used aminobisphosphonates to generate a predominantly Vδ2^+^ population (83, 87), a series of papers eventually demonstrated the possibility for expanding γδT cells with a broad range of γδTCR subsets using either plant-derived T-cell mitogens such as concanavalin A (88–90) or artificial antigen-presenting cells (aAPC) engineered to express co-stimulatory ligands and membrane-bound IL-15 (91). Two groups used a CD19^+^ aAPC system to expand Vδ2^−^ γδT cells, demonstrating that the repertoire of γδT cells produced could be influenced by the loading of anti-γδTCR antibodies to the CD64 expressed on the aAPC (37, 92). Furthermore, this approach can be used to specifically propagate anti-CD19 CAR^+^ γδT cells (93). Deniger and colleagues generated a CAR^+^ γδTCR^+^ population containing a broad range of Vγ and Vδ chain combinations using negative selection following CAR gene transfer to the whole peripheral blood mononuclear cell (PBMC) population. γδT cells were isolated on the day after electroporation and propagated on CD19^+^CD64^+^CD86^+^CD137L^+^IL-15^+^ aAPCs in the presence of IL-2 and IL-21; the aAPCs were refreshed weekly. The resultant γδT cell population showed low expression of exhaustion markers such as CD57 and contained a heterogeneous mixture of memory phenotypes. This expansion technique has been shown to preserve the distribution of Vδ1^+^, Vδ2^+^, and Vδ1^−^/Vδ2^−^ γδT cell subsets within a donor PBMC sample (37, 92). Singh et al. had previously demonstrated that culture using this aAPC system produced a selection pressure for CAR^+^ αβT cells (94) resulting in >90% CAR^+^ αβT cells after 28 days of co-culture, but this effect was muted when CAR^+^ γδT cells were expanded, presumably due to the inherent reactivity of non-transduced γδT cells against the aAPC leading to non-specific proliferation. aAPC based expansion may be particularly advantageous for γδT cells due to their expression of CD28 and CD137 which interact with CD86 and CD137L on the aAPC, and expression of CCR7 and CD62L by the CAR^+^γδTCR^+^ cells suggested that they had the capacity to home to the bone marrow and lymph nodes where CD19^+^ leukemia is known to reside. The CAR^+^ cells produced IFNγ, TNFα, MIP-1α, MIP1β, and RANTES following CAR activation through co-culture with a huCD19^+^ murine cell line which does not engage the γδTCR due to inter-species differences (95, 96), and killed human CD19^+^ cell lines with much greater efficacy than CAR^−^γδTCR^+^ cells (93). Immunodeficient mice xenografted with CD19^+^ffLuc^+^ NALM6 B-cell leukemia showed enhanced survival following CAR-γδT cell treatment compared to untreated, though a non-transduced or irrelevant CAR control was not included in the *in vivo* study so the *in vivo* activity is harder to dissect.

Engineering strategies which harness the innate properties of Vγ9Vδ2 T cells would seem to be the best justification for using them as an alternative “chassis” for CAR-T cell therapy. CARs were initially developed to bypass the αβTCR, limited as it is by MHC restriction and a requirement for specific TAA epitopes to be presented. The Vγ9Vδ2 TCR is not subject to these limitations; through its MHC-unrestricted detection of moieties associated with cellular stress. As such, there is an opportunity to “tune” the CAR-T cell response by modulating the level of stimulus delivered by the CAR. So far, this has been demonstrated in the context of neuroblastoma, against which Vγ9Vδ2^+^ T cells have minimal innate cytotoxicity (37), in part due to the tumor shedding soluble NKG2D ligands which block NKG2D activation (97, 98). If further stimulus is provided to the γδT cell, this cytotoxicity can be restored, either *via* a conventional second-generation CAR (90, 99) (Figure 1D), opsonization of the target cell (34, 37) or, as was recently shown, by restoring the NKG2D signal using a chimeric costimulatory receptor (CCR), that lacks CD3ζ, but contains the endodomain motif from the NKG2D adaptor, DAP10 (GD2-DAP10, Figure 1E). This approach enhanced killing of GD2^+^ neuroblastoma cells but did not induce cytotoxicity against GD2^+^ cells that did not engage the Vγ9Vδ2 TCR. Cytokine release was also controllable using this “AND gate” system; IL-2, IFNγ, and TNFα were only released from GD2-DAP10^+^Vδ2^+^ cells when they received both CD3 and CCR stimulus, whereas in GD2-28ζ^+^Vδ2^+^ cells, only CAR stimulus was required (99).

Chimeric costimulatory receptors have also been used in the context of αβT cells. They can deliver an isolated costimulatory signal to support antigen-specific proliferation (100), enhance tumor specificity by dividing CAR and CCR stimuli such that two antigens are required for activation (101), or reverse the suppressive effects of tumor PD-L1 through a PD1-CD28 chimeric receptor (102). In two of these studies, a separate CD3 signal was provided, either using OKT3 anti-CD3 (102), or a separate CD3ζ containing CAR (101). The earlier work by Krause et al. was particularly innovative; a chimeric anti-GD2 receptor with a CD28 endodomain supported antigen-specific proliferation of αβT cells in a TCR or CD3-dependent manner. When tested in the context of GD2^+/−^ tumor cells, they also confirmed that signal 1 could be provided by the TCR (100) and that their CCR would function under these conditions. The promiscuous, MHC-independent reactivity of γδT cells to danger-associated molecular patterns rather than MHC-restricted peptide epitopes could offer an opportunity to broaden this approach.

In addition to the possibility of avoiding on-target off-tumor toxicity, CAR expressing γδT cells retain the ability to antigen to cross-present (53, 90, 103–105). A recent study indicated that γδT cells transduced with second-generation anti-GD2 CARs (GD2-28ζ) retain the ability to cross-present TAAs leading to a clonal expansion of αβT cells. Using a 25 amino acid fragment of the melanoma antigen MART-1 which encompasses a 10 amino acid epitope but is too long to be MHC-presented in its un-processed form, Capsomidis et al. demonstrated that HLA-A201^+^V2δ^+^GD2-28ζ^+^ cells pulsed with the long peptide were able to elicit secondary expansions in αβT cells expressing a HLA-A201-restricted MART-1 αβ TCR (90). Vδ2^+^GD2-28ζ^+^ cells also retained the ability to migrate toward tumor cell lines; GD2-28ζ expression had no effect on migration in either Vδ1^+^ or Vδ2^+^ subsets in an *in vitro* trans-well assay. The next step in these investigations would be to show that γδT cells can cross-present antigens derived from cells that they have themselves killed. If this were successful it would raise the possibility that the broad anti-tumor reactivity of γδT cells could be used to prime a diverse population of autologous αβT cells against many tumor-derived antigens simultaneously. Demonstration of enhanced anti-tumor activity by “γδT-cell primed” αβT cells would further validate this approach.

## Transduction Strategies for γδT Cells

When engineering γδT cells it is important to select appropriate tools. In general, long-lasting transduction strategies which work well for αβT cells work well for γδT cells also. The predicted shorter lifespan of infused γδT cells offer the opportunity to use more transient engineering approaches as well, as the infused cells may not persist in the host long-term.

Many groups continue to use gammaretroviral vectors for transducing γδT cells. High transduction efficiencies are achievable using a Maloney murine leukemia virus-based vector, SFG (87), pseudotyped with the envelope of the feline endogenous retrovirus RD114 (99), or gibbon-ape leukemia virus envelopes. Gammaretroviral transduction has the advantage of allowing preparation of large, high titer batches of virus because of the availability of packaging cell lines which can be stably transduced to produce virus containing the construct of interest (106). Gammaretroviruses, lacking the machinery to penetrate the nucleus, require the cells to be actively cycling in order to achieve transduction as viral nucleic acids can enter through nuclear pores (107). This is not a restriction in the engineering of αβT cells, and the specific and rapid expansion of Vγ9Vδ2^+^ T cells in response to aminobisphosphonates allows for similar strategies to be applied. Transduction of other γδT cell subsets using gammaretroviruses following concanvaulin A driven expansion is less predictable, however, with variable yield (90). There has been some concern regarding the potential for insertion-site-mediated mutagenesis following gammaretroviral gene transfer (108), which has prompted some in the field to favor lentiviral vectors which have a safer insertional profile (107). There is little published data to compare lentiviral transduction techniques for γδT cells, though one group did find that the use of a vesicular stomatitis Indiana virus G-protein containing envelope in combination with a simian immunodeficiency virus transfer vector consistently provided higher transduction efficiency than a human immunodeficiency virus-based vector with the same envelope (transduction efficiency 65% vs 42%, *p* = 0.04) (109). New genome editing technologies, such as CRISPR-CAS, allow targeting integration of viral vectors with several potential advantages including avoidance of integration into oncogenic loci, and integration into loci that optimize CAR or TCR expression (11, 110).

Non-viral methods of transduction have provided particular advantages when engineering γδT cells. The Sleeping Beauty Transposon system (111, 112) uses enzymes originally derived from fish to insert new genetic material into host cells. It has not yet been established that the SB-transposon system is more efficacious than lentiviral transduction, and there is an unknown potential for insertional mutagenesis (113). Cells must be electroporated in order for gene transfer to occur, but do not require a specific proliferative stimulus. As described above, Deniger et al. used this to good effect to express an anti-CD19 CAR (CD19RCD28) in a polyclonal repertoire of γδT cells which were subsequently expanded using CD19^+^ aAPCs (91, 93). Unlike proliferation-driven transduction techniques, there was no “skewing” of the γδT cell population toward a particular Vγ/Vδ subset. This may be of particular interest if engineered γδT cells were to be directed against epithelial tumors, as non-Vδ2^+^ γδT cells are enriched in epithelial surfaces, a tropism which could be harnessed.

Viral or transposon-based gene transfer generates stable construct expression over time by integrating into the transduced cell genome. This has been considered important in the CAR-T cell field as it allows for persistence of CAR-T cells for weeks or months. More transient CAR expression strategies have been suggested as a means of reducing toxicity following CAR-T cell infusion. mRNA transfection using electroporation was used to generate γδT cells expressing NKT-cell derived TCRs (79) and, more recently, HLA-A2/gp100-specific TCR or CARs targeting melanoma-associated-chondroitin-sulfate-proteoglycan (MCSP). MCSP is a tumor-associated antigen expressed on melanoma (114), glioma (115), triple-negative breast cancer (116), and sarcomas (117). Expression peaked at around 24 h after transfection, returning to baseline by around 72 h (77). Similar techniques have been tested clinically in the context of CAR^+^ αβT cells, where they were used to transfect cells with a construct targeting mesothelin (118), though repeated infusions of CAR-T cells were required, presumably because of the need to “top-up” the reservoir of circulating CAR-T cells as expression was lost.

## Concluding Comments

While there is promising data to suggest that gene-modified γδT cells may be an attractive candidate for clinical studies, the bulk of enthusiasm in the cellular immunotherapy field focuses on αβT cells. Undoubtedly, more data are available on αβT cell engineering, increasing the likelihood of introducing novel constructs to the clinic. γδT cells appear to cause less graft-versus-host disease than αβT cells while retaining graft-versus leukemia activity in the hematopoietic stem-cell transplant setting (119). *In vivo* data on the function of CCR expressing γδT cells and/or the antigen cross-presentation capacity of γδT cells are not yet available. Reduced toxicity and the potential for antigen cross-presentation are compelling arguments for the potential benefits of γδT cells over αβT cells as a substrate for CAR expression. The difficulty of modeling the more subtle aspects human γδT cell activity in a murine system makes this data particularly hard to generate. However, within the increasingly crowded field of cancer immunotherapy, orthogonal approaches to cellular engineering are required to move the field forward. The challenges of off-tumor toxicity, poor penetration of solid tumors and tumor immune evasion need to be addressed. It is no surprise that CD19 CAR-T therapies have been more successful than others, depletion of healthy CD19^+^ B-cells is considered an acceptable toxicity and the disease resides in the hematological compartment. In other cases, on-target off-tumor toxicity has been severe or fatal, experiences which have shaped the way that target antigens are chosen (20, 21). Investigating the potential of alternative CAR “chassis” to harness the innate characteristics of particular cell types factorially increases the number of options available. As cells sharing adaptive properties of conventional αβT-cells and innate properties of NK-cells, γδT cells are a highly attractive and potentially efficient candidate for this process of optimization.

## Author Contributions

All authors listed have made a substantial, direct, and intellectual contribution to the work and approved it for publication.

## Conflict of Interest Statement

JA holds stock in Autolus PLC and JA and JF perform consulting work for TC Biopharm.
